# Non-Carcinogenic Health Risk Evaluation of Elevated Fluoride in Groundwater and Its Suitability Assessment for Drinking Purposes Based on Water Quality Index

**DOI:** 10.3390/ijerph19159071

**Published:** 2022-07-25

**Authors:** Zahid Ullah, Yifan Xu, Xian-Chun Zeng, Abdur Rashid, Asmat Ali, Javed Iqbal, Mikhlid H. Almutairi, Lotfi Aleya, Mohamed M. Abdel-Daim, Muddaser Shah

**Affiliations:** 1 State Key Laboratory of Biogeology and Environmental Geology, School of Environmental Studies, China University of Geosciences, Wuhan 430074, China; zahid_environ225@cug.edu.cn (Z.U.); xuyifancug@163.com (Y.X.); abdur.rashid@bs.qau.edu.pk (A.R.); asmat@cug.edu.cn (A.A.); javediqbal@cug.edu.cn (J.I.); 2 Department of Zoology, College of Science, King Saud University, P.O. Box 2455, Riyadh 11451, Saudi Arabia; malmutari@ksu.edu.sa; 3 Chrono-Environnement Laboratory, UMR CNRS 6249, Bourgogne, Franche-Comté University, CEDEX, F-25030 Besancon, France; lotfi.aleya@univ-fcomte.fr; 4 Pharmacology Department, Faculty of Veterinary Medicine, Suez Canal University, Ismailia 41522, Egypt; adeldaim.m@vet.suez.edu.eg; 5 Department of Botany, Abdul Wali Khan University, Mardan 23200, Pakistan; muddasershah@awkum.edu.pk; 6 Natural and Medical Sciences Research Center, University of Nizwa, P.O. Box 33, Birkat Al Mauz, Nizwa 616, Oman

**Keywords:** groundwater fluoride, geochemical modeling, health risk assessment, source provenance, Quetta Valley

## Abstract

Fluoride (F^−^) contamination in drinking groundwater is a significant human health risk in Pakistan. Moreover, high fluoride pollution in drinking water causes a variety of disorders, including dental, neurological, and skeletal fluorosis. The aim of this research was to evaluate the health risk of elevated fluoride in groundwater and its suitability assessment for drinking purposes. The total of (*n* = 37) samples were collected from community tube wells of Quetta Valley, Balochistan, Pakistan. The results show a mean pH value of 7.7, TDS of 404.6 mg/L, EC of 500 µs/cm, depth of 96.8 feet, and turbidity of 1.7 nephelometric turbidity units. The mean values of HCO_3_^−^, Ca^2+^, Mg^2+^, and Na^+^, were 289.5, 47.5, 30.6, and 283.3 mg/L, respectively. The mean values of SO_4_^2−^, NO_3_^−^, K^+^, Cl^−^, and Fe^2+^, were 34.9, 1.0, 1.6, 25.6, and 0.01 mg/L, respectively. The F^−^ concentration in the groundwater varied between 0.19 and 6.21, with a mean value of 1.8 mg/L, and 18 samples out of 37 were beyond the WHO recommended limit of 1.5 mg/L. The hydrochemical analysis results indicated that among the groundwater samples of the study area, 54% samples were Na-HCO_3_ type and 46% were mixed CaNaHCO_3_ type. The saturation indices of the mineral phases reveal that the groundwater sources of the study area were saturated with CaCO_3_ and halide minerals due to their positive (SI) values. Such minerals include calcite, dolomite, gypsum, and fluorite. The principal component analysis results reveal that the groundwater sources of the study area are contaminated due to geological and anthropogenic actions. The health risk assessment results of the F^−^ concentrations show the ranges of ADD_ingestion_ for children, females, and males in the Quetta Valley, and their mean values were observed to be 0.093052, 0.068825, and 0.065071, respectively. The HQ_ingestion_ mean values were 1.55086, 1.147089, and 1.084521 for children, females, and males, respectively. It was noticed that children had the highest maximum and average values of ADD_ingestion_ and HQ_ingestion_ in the research area, indicating that groundwater fluoride intake poses the greatest health risk to children. The water quality index (WQI) analyses show that 44% of the samples belong to the poor-quality category, 49% were of good quality, and 8% of the samples of the study area belong to the excellent category.

## 1. Introduction

Fluoride (F^−^) is an important mineral for humans, as it helps to enhance teeth and skeletal tissues [1]. However, fluorosis is a disease that destroys the teeth and bones and is caused by ingesting too much fluoride, which is widely found in drinking/groundwater (GW) [2]. Moderate doses have dental effects, but long-term ingestion can lead to serious bone disease [3]. Furthermore, prolonged exposure to F^−^ toxins can harm the reproductive, neurological, musculoskeletal, developmental, endocrine, and renal systems, leading to genotoxicity in some cases [4]. F^−^ is fluorine’s major inorganic toxicant, which is favored by an alkaline pH, high sodium cations (Na^+^), higher bicarbonate (HCO_3_^−^), and low calcium (Ca^2+^) ion concentrations [5]. F^−^ enrichment can be found in a variety of habitats, including GW, soil, rocks, food, air, flora, fauna, and the human body [6]. GW is a key source of F^−^ for human ingestion, possibly recognized and controlled by hydrogeology, climatic factors, anthropogenic actions, and the regional chemistry of the host rocks [7]. Soil contains 0.3 g/kg F^−^ content in Earth’s crust in terms of natural abundance, and it is the 13th most important source [8]. GW (F^−^) concentrations are generally higher in discharge zones, especially where average or shallower groundwater depths are found [9].

Fluorite, fluorspar, fluorapatite, topaz, hornblende, tourmaline, villiaumite, amphiboles, mica, biotite, and muscovite are the minerals that contain F^−^ [10]. Aside from these minerals, igneous and sedimentary rocks, as well as some weathering silicates, provide a large amount of F^−^ to GW [11]. F^−^ concentrations are also found in various types of rocks, such as basalt (20–1060 ppm), granite (20–2700 ppm), shale and clay (10–7600 ppm), limestone (0–1200 ppm), sandstone (10–880 ppm), phosphorite (24,000–41,500 ppm), and coal (40–480 ppm), with average values of (360), (870), (800), (220), (180), (31,000), and (80 ppm), respectively [12]. Food, water, industrial exposure, pharmaceuticals, cosmetics, and mining activities are all common ways for F^−^ to permeate the environment and humans [13]. F^−^ concentrations in GW that are too high are considered a severe health risk [14]. Fluorosis is a widespread endemic disease that has a geological basis. Indeed, there is a well-established relationship between the severity of fluorosis and (F^−^) concentrations in GW [15]. In terms of a permissible level of (F^−^) in groundwater, the World Health Organization (WHO) recommends 1.5 mg/L [16]. Highly prevalent fluorosis affects 260 million people in 25 countries globally, with 100 million in Southeast Asia, including India, Pakistan, and Sri Lanka [17,18,19]. Fluorosis, both teeth and skeletal, is a worldwide issue that has been established in recent research studies [20]. The overuse and continuous drinking of GW for domestic reasons occur primarily in semiarid and rural parts of Pakistan, potentially causing the GW quality to deteriorate [21,22]. Conversely, in developing countries around the world, such as Pakistan, India, and China, growing industrialization, mining, and urbanization are serious environmental concerns [23]. In Pakistan, GW is used for domestic, agricultural, and industrial reasons. As a result of the use of groundwater for various purposes, the quality and quantity of GW have deteriorated. F^−^ contamination of GW sources in Pakistan has been documented in many regions, such as Dargai [23], Negar Parkar [24], Sialkot [25], UmarKot [26], Nagar Parkar [27], Swat [1], and Peshawar; however, the information on F^−^ contamination in GW sources of Pakistan is still limited. To investigate the real situation concerning F^−^ pollution, it was compulsory to carry out a detailed survey of GW sources in Pakistan.

As mentioned above, we studied the F^−^ concentrations in the community tube well water of Quetta, Pakistan. This study, in which we investigated the F^−^ concentrations in the community tube wells throughout the whole valley, was the first to be conducted in the study area. The local peoples of the area use tube well water for domestic and agricultural practices; however, the tube well water samples were observed to have elevated concentrations of F^−^ contamination. The aim of this research was (1) to investigate the physicochemical features of the tube well water, (2) to investigate the study area’s particular distribution pattern and the amount of fluoride risk to the local population; (3) to investigate the relationship between fluoride and other groundwater variables, as well to identify pollutant sources; (4) to investigate the geochemical mechanisms of fluoride enrichment in the aquifer system of the study area; and (5) to evaluate the suitability of the tube well water for ingestion using the WQI approach.

## 2. Study Area

### 2.1. Geography and Geology

**Figure 1:**
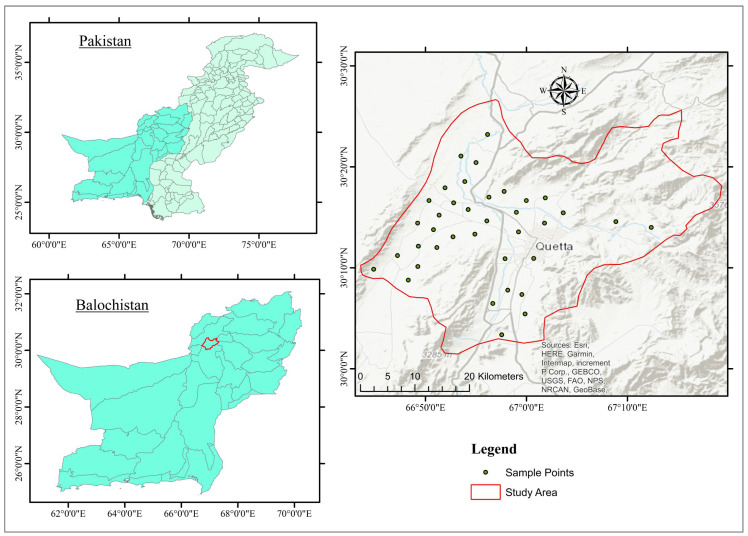
Location of the study area and groundwater sampling stations.

The research area, Quetta Valley, is the provincial capital of Baluchistan, Pakistan, and is situated in the western highlands of the province between latitudes of 30°00′ and 30°20′ and longitudes of 66°50′ and 67°15′ as a landlocked watershed sub-basin, as shown in Figure 1 [28]. The research was performed in the month of April 2021 in the provincial capital Quetta, which is connected in the west to Afghanistan. Quetta is surrounded on all sides by high mountains, including Chilton, Takatu, and Murdar, with heights ranging from 1000 to 4000 m [29]. The climate is primarily sandy, with long, cold winters and short, warm summers. Due to large folds and faults, the structural history of Quetta and its vicinity is highly complex, as it marks the western edge of the collision zone between the Indo-Pakistan and Eurasian plates, which absorbed the Tethys Ocean. Geological structures from the recent Quaternary to the Jurassic era can be found in the study region (Quetta Valley). Weathering from the surrounding geological formations formed unconsolidated (sand, silt, and clay) to semi-consolidated (claystone, sandstone, and subordinate conglomerate overlying calcareous and carbonaceous strata), and quaternary deposits covering much of the basin. Middle Jurassic, Chilton limestone that is light to dark grey, black, and brownish to bluish grey and huge white limestone that is fine-grained, oolitic, and reefoid, were discovered in the Quetta area and are in the form of rocks in the east and west of the valley, with the largest thickness up to 1800 m [30]. The Quetta area contains Lower Jurassic rocks with a thickness of up to 1800 m. Grey to dark grey, thin- to medium-bedded coarse-grained shelly, oolitic, pesolitic, and pellitic limestone is interbedded with shale and sandstone at the base of the Lower Jurassic. Chocolate brown or dark grey limestone from the Tertiary to Cretaceous is exposed in the southeast and southwest, with thicknesses ranging from 25 to 60 m. The lower tertiary Eocene coastal shelf sequence is made up of shale with interbedded sandstone and has a thickness of 915 m in the northeast. Shale with interbedded limestone and an approximate thickness of 130 m can be found in the Upper Jurassic and Cretaceous (Kjm); however, it narrows near Quetta [31].

### 2.2. Hydrogeology and Lithology

**Figure 2:**
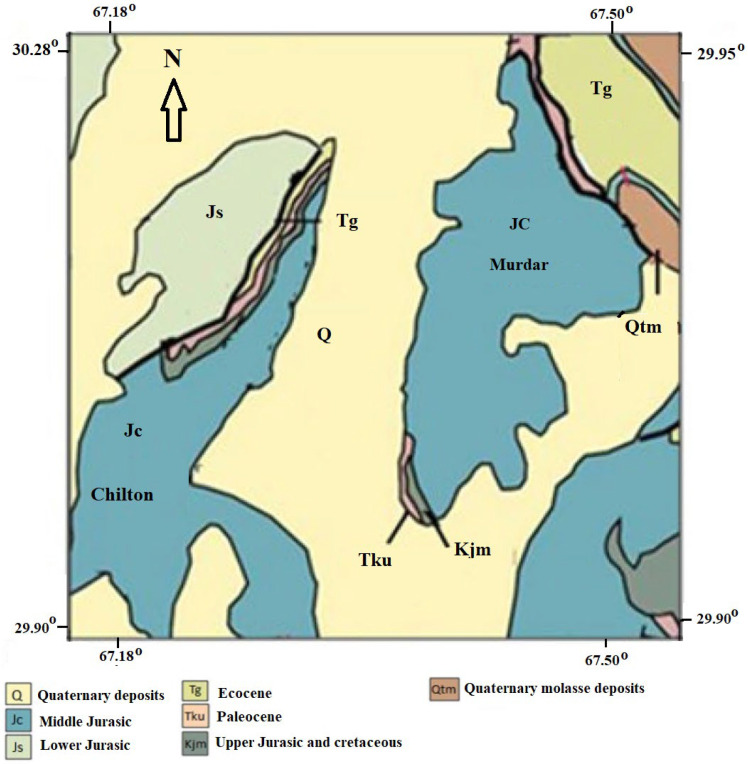
Geological map of the study area.

Karezes and springs were the primary irrigation sources in the upland districts of Baluchistan in the early twentieth century (60%). Groundwater is currently the only available source in the Quetta Valley, and residents use it for agriculture, industry, and domestic purposes. The water table in the Quetta Valley’s aquifer system is rapidly falling as a result of overexploitation, and thus, the groundwater is under severe stress. In different areas of the Quetta Valley, the groundwater decline ranged from 2.8 to 30.66 m between 1987 and 2013 [32]. In the Quetta Valley, there are two types of aquifers: unconsolidated alluvial aquifers and hard bedrock aquifers. The bulk of GW is collected from thick (30–900 m) Quaternary alluvial deposits (containing different proportions of gravel, sand, and silt) in the main valleys, as shown in Figure 2, while less GW is extracted from Jurassic bedrock aquifers. In the foothills and surrounding mountain areas, these formations are exposed or hydraulically connected, and the aquifers are refilled by the infiltration of precipitation runoff. The primary recharging zones are the piedmont zone and stream beds; the gravel in these zones slopes down into the valley and is buried beneath silt and loess that can be 100–200 feet thick in some places [32]. Rainwater infiltrates basin piedmont and gravels to recharge the aquifers in the central plain, passing through a variety of lithological layers. The velocity of movement is determined by the size, configuration, and gradient of the rock openings, as well as the lithological unit with which it comes into contact [33].

## 3. Material and Methods

### 3.1. Sampling and Analysis

In the Quetta Valley, GW samples (*n* = 37) were collected from community tube wells to determine the fluoride concentrations and other physicochemical parameters. The samples were collected randomly to cover the whole valley. The sampling survey was performed in the month of April 2021. Before sampling, the community tube well pumps were started for 10–15 min to avoid the effects of stagnant water. Groundwater samples collected for major cations were filtered through Whatman filter paper (No. 0.42 µm) to protect not only the atomic absorption spectrophotometer (APHA, 1998), but also to confirm its accuracy, following the method of [34]. The samples were kept in polyethylene bottles that had been thoroughly cleaned and soaked twice in deionized water. The GW samples (tube wells) were collected using a duplicate sampling approach. The basic water quality parameters, such as pH, electrical conductivity (EC), and total dissolved solids (TDS) in the GW samples, were measured in situ with a portable Hanna apparatus that had been calibrated before use [35]. After immediately transporting the samples to the Hydrogeochemistry Laboratory, Quetta, the alkalinity was determined using the titrimetric method. The sulfate (SO_4_^2−^) and nitrate (NO_3_^−^) concentrations were determined with a UV visible spectrophotometer (DR 5000) using a conventional turbid metric method at wavelengths of 420, 410, and 690 nm, respectively [36]. The contents of chloride (Cl^−^) and fluoride (F^−^) were determined using the “Mohr’s technique and Fluoride Analyzer” ion-selective electrode (ISE) (HANNA Instruments, Japan, Model No. HI 5222 and HI 4110, type Solid-state; Combination) [37]. The principal cations in the GW samples, including Ca^2+^, magnesium (Mg^2+^), sodium (Na^+^), and potassium (K^+^), were measured using a flame atomic absorption spectrophotometer (Varian Spectra AA–240, Australia) under standard operating settings [38]. To assess the analytical precision and correctness of the GW data, the ionic charge balance of cation and anion errors (*ICBE*) was determined. The consistency and validity of the water quality were determined by the ionic charge balance error (*ICBE*), in which the total sum of all anions is subtracted from the total sum of all cations minus, then divided by the total sum of all cations plus (+) anions, and then multiplied by 100 to attain the percentage contributions of the groundwater samples. As a result, all of the groundwater samples were found to be within the ±5% range using Equation (1). As a result, the results of the chemical analyses were accurate.

(1) $$ICBE=\frac{\left[\;\sum cations-\sum anions\right]}{\left[\;\sum cations+\sum anions\right]}\times 100$$

Milliequivalent per liter (meq/L) is the unit of measurement for ionic absorption. Following an established protocol, only samples with less than ±5% CBE were approved for further examination.

### 3.2. Quality Assurance and Quality Control

Routine quality control checks, standardized operating protocols, reagent blanks, standard calibration, and duplicate analyses were performed to achieve accuracy and precision in the results of the analytical data. The chemicals used for analysis were obtained from Germany (Merck Company, Kenilworth, NJ, USA). To remove the contaminants, all glassware was thoroughly washed with deionized water and a 30% HCl solution. Glassware was oven-dried after being washed following the standard protocol adapted from [39].

### 3.3. Statistical Analysis

#### 3.3.1. Principal Component Analysis

Principal component analysis (PCA) is a technique for reducing the dimensionality of data while maintaining as much of the information contained in the original data as feasible. PCA does this by projecting data onto a lower-dimensional subspace that retains the majority of the variance between data points. PCA is a widely used technique for reducing data complexity. PCA can maintain the majority of the data while lowering the complexity of the data [40]. The sources of pollution were determined using principal component analysis (PCA) in this study. PCA was performed using SPSS Software (Version 20.0).

#### 3.3.2. Correlation Analysis

A statistical approach for detecting how closely two variables is related is correlation analysis. Positive values represent that the water variable was significantly influenced by different processes, whereas negative values indicate that the saturation of the water chemistry was not affected. Thus, a Pearson correlation coefficient (r) significant pair at a 0.05 alpha level with 95% confidence was calculated [41]. In this work, Pearson correlation analysis was employed to determine the relationship between numerous water chemical characteristics.

#### 3.3.3. Saturation Indices

Saturation indices can be used to determine the tendency of GW to dissolve or precipitate a specific mineral. Furthermore, measuring the mineral balance helps in estimating the dissolved mineral reactivity in water. PHREEQC Interactive, a geochemical simulation tool (version 3.4), was used to calculate the saturation indices.

#### 3.3.4. Water Type

Piper diagrams are often used to describe the hydrogeochemical types and relative concentrations of major anions and cations in different samples, and they can also highlight certain potential geochemical processes that can help with groundwater quality knowledge and assessment. A Piper diagram was built using Grapher (version 14).

#### 3.3.5. Water Quality Analysis for Drinking

Weighted arithmetic water quality index (WAWQI) values were used to determine the appropriateness of the groundwater for drinking. The WAWQI values were calculated using the WHO (2011) drinking water standard, following Equation (2).

(2) $$WQI=\sum_{i=1}^{n}SIi$$

where *SIi* and *W_i_* are the sub-index and relative weight of the *i*th parameter, respectively, qi is the rating based on the concentration of the *i*th parameter, and (*n*) refers to the number of parameters.

#### 3.3.6. Health Risk Assessment

The oral pathway’s daily intake (*EDI*) and hazard quotient (HQ) due to fluoride were calculated using USEPA guidelines (1992).

(3) $$EDI_{oral}=\frac{C\times IR\times EF\times ED}{BW\times AT}$$

where *C* is the concentration of dissolved F^−^ in groundwater (mg/L), *IR* is the intake of water per day (L Day ^−1^), *EF* is the exposure frequency (days y^−1^), *ED* is the exposure duration (y), *AT* is the averaging time (days), and *BW* is the body weight. The product of *EF* and *ED* is equal to that of the *AT*, and thus, Equation (3) can be further simplified to *EDI_oral_* = *C* × *IR*/*BW* (3a).

HQ_ORAL_ = EDI_ORAL_/RfD_ORAL_ (4)

An HQ_ORAL_ < 1 specifies that it is safe to consume the water, while an HQ_ORAL_ > 1, indicates potential health effects on human health in the form of fluorosis. Fluorosis has a negative impact on human health. The reference oral dose, RfD_ORAL_, was calculated to be 0.06 mg F^−^ day^−1^ of water and kg^−1^ body weight. For adults, a water intake rate of 2 L Day^−1^ and body weight of 70 kg were used, while the corresponding values were 0.89 L Day^−1^ and 15 kg for the case of children.

## 4. Results and Discussion

### 4.1. Groundwater Composition

Table 1 shows the geochemical compositions of GW in the form of the minimums, maximums, means, and standard deviations. The pH value of groundwater samples ranged from 7.1 to 8.1 with a mean value of 7.7, suggesting that the GW is slightly alkaline. Slightly acidic conditions of water may occur when it combines with carbon dioxide during the process of precipitation. The pH is a key water quality parameter and its determination is compulsory due to its vital role in the saturation of GW variables [42]. However, GW in an acidic medium (pH = 6) may increase the solubility of metal ions. The variability of pH in groundwater causes variation in the chemical composition of groundwater [43]. The TDS values ranged from 290 to 594 mg/L, with an average value of 404.6 mg/L. All of the samples under the WHO recommended values of 1000 mg/L. Water containing more than 1000 mg/L dissolved solids has an unpleasant odor and is unfit for drinking. The flavor, hardness, and corrosive qualities of water are all affected by high TDS levels [44]. The value of the electrical conductivity (EC) varied between 261 and 705 µs/cm, with an average value of 500 µs/cm. The value of EC was beyond the acceptable limit of 400 µs/cm recommended by WHO. The EC was directly related to the GW ion concentrations, resulting in greater salinity and dissolved concentrations [45]. The depth of the tube wells in the study area was in the range of 70–135 (feet), with an average value of 96.8 (feet). The turbidity values varied between 0.5 and 3.7, with an average value of 1.7 nephelometric turbidity units (NTU), and all of the samples were within the acceptable limit recommended by WHO. However, surface recharge, water runoff, weathering processes, and industrial effluents can all contribute to increased turbidity in the area’s water system. Furthermore, excessive turbidity can be caused by shallow and poorly designed wells [46]. The value of HCO_3_^−^ varied between 143 and 532 mg/L, with an average value of 289.5 mg/L, and was beyond the permitted limit of WHO. The dissolution of calcite, carbonate, marble, and dolomite-bearing minerals causes an increase in HCO_3_^−^ concentrations [47]. The values of Ca^2+^ and Mg^2+^ were 17–78 mg/L and 15–73 mg/L respectively, with average values of 47.5 and 30.6 mg/L, respectively, and within the WHO permitted limit. Elevated concentrations of Ca^2+^ and Mg^2+^ in GW are due to rock/water interaction [48]. The values of Na^+^ in the GW samples varied between 135 and 549 mg/L, with an average value of 283 mg/L, and were higher than the permitted limit recommended by WHO. However, salt deposit erosion and Na^+^-bearing rock minerals, such as halite (NaCl), and plagioclase (NaAlSi_3_O_8_-CaAl_2_Si_2_O_8_) minerals are the most common causes of higher Na^+^ levels in GW [49]. The values of SO_4_^2−^ and NO_3_^−^ in the GW samples varied between 13 and 90 mg/L and 0 and 4 mg/L, respectively, and their mean values were 34.9 and 1.0 mg/L, respectively. High concentrations of SO_4_^2−^ in GW sources is due to mines and smelters, as well as kraft pulp and paper mills, textile mills, and tanneries, which may release sulfates into water [50], while elevated concentrations of NO_3_^−^ in GW are due to human activities, such as agriculture, industry, home effluents, and combustion engine emissions, which cause nitrate concentrations to rise [51]. The value of K^+^ varied between 1 and 4 mg/L, with an average value of 1 mg/L. All of the samples were within the WHO permitted limit of 10 mg/L. The values of Cl^−^ and Fe^2+^ were in the ranges of 12–89 mg/L and 0.01–0.2 mg/L, respectively, with average values of 25.6 mg/L and 0.01 mg/L. However, chlorine is found in a variety of minerals found in common rocks, and its release into water as chloride ions is often delayed and occurs by mechanisms other than dissolution [52], while the source of iron (Fe^2+^) in GW is mostly attributed to geogenic processes, such as lateral weathering processes and corrosion products that release iron into water [20].

### 4.2. Fluoride Contamination in Groundwater

Fluoride values in the study region ranged from 0.19 to 6.21 mg/L, with an average value of 1.8 mg/L, as shown in Table 1. Out of the total number of samples (*n* = 37), 18 samples (49%) were beyond the WHO recommended values, while 51% samples were inside the acceptable limits. F^−^ in groundwater is controlled by many factors, such as cation exchange, evaporation, higher concentrations of HCO_3_^−^ and Na^+^, base ionic exchange, and residence time [20]. The weathering of rock is recognized as the major control mechanism to blame for elevated fluoride concentrations in the study area’s groundwater [4]. Moreover, base ion exchange mechanisms show that greater Na^+^ and HCO_3_^−^ concentrations play a role in the activation of Na^+^ and F^−^ in groundwater via displacement reactions and the common ion effect [13]. Researchers also found that a higher retention time (HRT) is one of the likely causes of increasing F^−^ levels in groundwater [16]. The occurrence of high F^−^ concentrations in groundwater depends on numerous factors, such as the saturation state with respect to fluorite. Moreover, the “common ion effect”, with respect to carbonate minerals, (e.g., calcite), indirectly controls F^−^ hydrogeochemical behavior. The Na-HCO_3_ water type saturated with calcite often has low concentrations of dissolved Ca^2+^ and higher F^−^ concentrations [53]. Moreover, granite rocks are rich in fluorite, and mica minerals further increase the F^−^ concentrations in the groundwater wells [54,55,56]. The following reactions explain the geological and geochemical behavior of water systems (Equations (5)–(7)).

2CaF_2_ + 4OH → 2Ca(OH)_2_ + 2F (5)

Muscovite: KAl_2_ (AlSi_3_O_10_)(F_2_, 2OH) → KAl_2_ (AlSiO_3_O_10_) (OH)2 + 2F (6)

Biotite: KMg3(Al_3_SiO_10_) (F_2_ + 2OH) → KMg3(Al-Si O) (OH)2 + 2F (7)

### 4.3. Hydrochemical Facies

**Figure 3:**
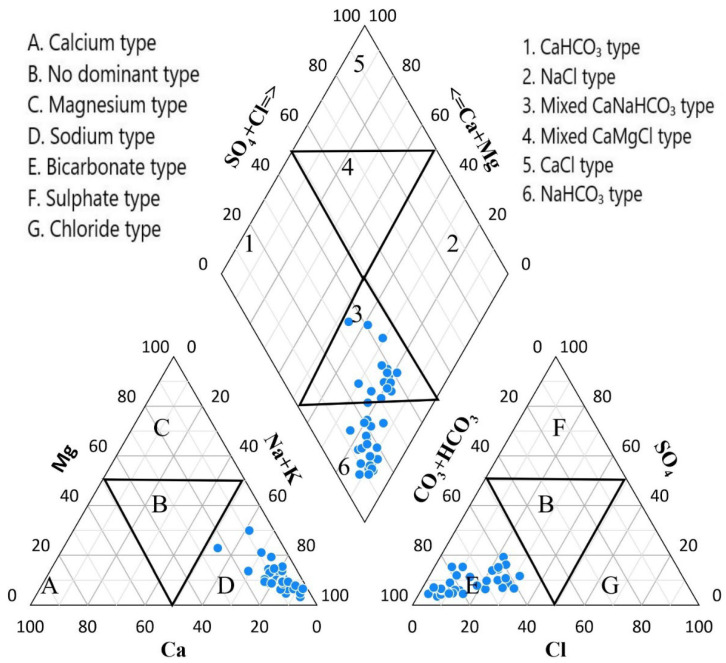
Piper diagram showing water type of the samples collected from community tube wells. The blue dots represnts the samples.

The entire phase of groundwater within a lithological structure is depicted by hydrochemical facies. A Piper diagram is quite crucial to understanding the evolution and flow pattern of GW. It is a graphical representation that shows the hydrochemistry of samples and their hydrochemical regimes. The levels of fluoride in an aquifer system mostly depend on the chemical features of the groundwater, which are identified by its hydrochemical facies. To illustrate the chemical differences among the GW samples, the samples were plotted on a Piper diagram, as shown in (Figure 3). In the current research work, 54%of the samples are of the NaHCO_3_ type, and 46% of the samples are of the mixed CaNaHCO_3_ type. This categorization can be accredited to mineral solubilization, rock/water interaction, and ion exchange processes. Our findings are supported by [1,7], who observed a higher F^−^ concentration associated with the NaHCO_3_ and mixed CaNaHCO_3_ water types in the underlying geological environment. With respect to cations, all of the samples were of the Zone D sodium type, which indicates the significance of silicate weathering, while the anions all of the samples were of the Zone E bicarbonate type, indicating the eminence of carbonate weathering in the study area [24].

### 4.4. Mineral Phases of Groundwater

**Figure 4:**
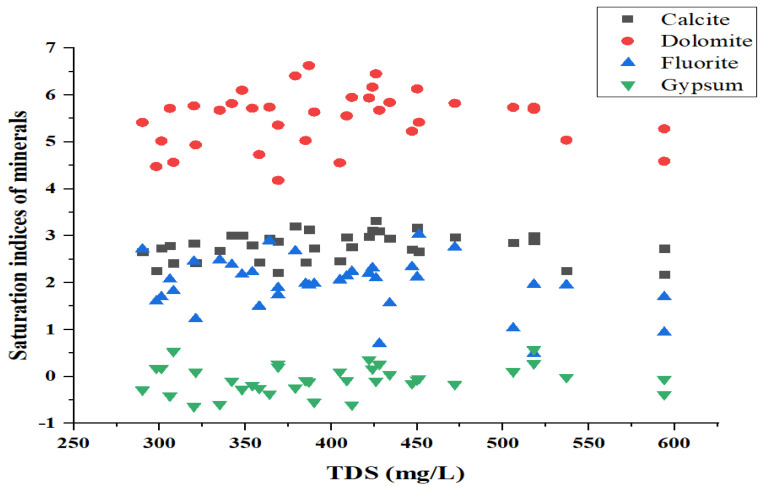
Saturation index of mineral phases in the groundwater samples of the study area.

Saturation measurements can be used to estimate subsurface minerals. Some minerals are found in equal concentrations in underground and surface waters. In the current investigation, SI calculations revealed that the carbonate and fluorite minerals had various degrees of saturation. The SI values of calcite, dolomite, fluorite, and gypsum, as shown in Figure 4, ranged from 2.2183 to 3.3232, 4.7319 to 6.6256, 0.4826 to 2.7129, and −0.0951 to 0.2794, respectively. These minerals may precipitate if SI > 1, and they may also dissolve if SI < 0. The gypsum value, on the other hand, was less than zero, indicating an unsaturated state, and the findings also suggest that Na^+^ and Cl^−^ may not be the primary sources of halite. This study found that silicate and fluorite minerals contributed to groundwater pollution in the studied area.

### 4.5. Principal Component Analysis

Table 2 shows the findings of principal component analysis (PCA) for groundwater parameters. After varimax rotation, the PCA results were obtained to elucidate the obtained components that impacted the groundwater. A total of five main factors were derived for groundwater parameters: F1, F2, F3, F4, and F5, with eigenvalues of 3.75, 2.0, 1.84, 1.34, and 1.20, respectively, resulting in total variances of 24.98%, 13.35%, 12.27%, 8.91%, and 8.0%, respectively.

Factor F1 was calculated to have a total variance of 24.98%, an eigenvalue of 3.75, and strong positive and negative loadings for groundwater variables HCO_3_^−^, Ca^2+^, Na^+^, and F^−^, the values of which were calculated to be 0.96, −0.68, 0.91, and 0.92, respectively. In the PCA results, the F1 results show that natural processes, such as carbonate (CaCO_3_) weathering, and the dissolution of aluminosilicate minerals, such as feldspar, biotite, fluorite, muscovite, calcite, and dolomite, control groundwater chemistry. Furthermore, the carbonate dissolution and biological decomposition of organic materials are the main factors that promote HCO_3_^−^ in GW. The efficiency of Na^+^ and HCO_3_^−^ in relation to fluoride reveals that alkaline climatic conditions facilitate the dissolution of fluoride [57]. Thus, the F1 results reflect a geogenic source of contamination in the GW sources of the study area. The F2 variability was calculated to be 13.35%, with an eigenvalue of 2.0 and showing strong loadings for the pH, TDS, and EC, the correlation coefficients of which were calculated to be 0.57, 0.52, and 0.55, respectively. The source of the TDS and EC in the GW sources is the effect of erosion of rocks with sulfide strata. Furthermore, high TDS levels in the GW showed ion dissolution, which might be attributed to gradually decreasing salts and minerals over time. The higher pH in the GW indicates that it is highly alkaline. Chemicals, minerals, pollutants, soil or bedrock composition, and any other contaminants that interact with a water supply may cause an imbalance in the water’s natural pH (=7) [55].

F3 was calculated to have a total variance of 12.27%, an eigenvalue of 1.84, and shows strong loadings for TDS, Mg^2+^, SO_4_^2−^, and Cl^−^, with coefficient r values of 0.60, 0.54, 0.81, and 0.51, respectively. The strong and moderate positive loadings of these parameters show that both anthropogenic and geogenic processes can play a vital role in the contamination of GW [15]. Factor F4, with a variance of 8.91% and an eigenvalue of 1.34, shows moderate loadings for turbidity and NO_3_^−^, with coefficient r values of 0.57, and 0.52, respectively. Sediment is frequently at the top of the list of compounds or pollutants that cause turbidity. Any watershed, however, has various sources of contaminants or physical factors that can change water clarity. These are classified as natural or background sources and human-induced sources. Erosion from upland, riparian, stream bank, and stream channel areas can all be natural sources. The source of SO_4_^2−^ in the GW is due to is sulfide weathering, which accounts for around half of the total sulfate. Rainfall is the second most significant source of sulfate input, with a 30% average contribution [42]. Thus, F4 reflects a mixed type of sources, which may include both natural and anthropogenic sources. F5 was calculated to have a total variance of 8% and an eigenvalue of 1.2 and shows a strong negative loading for depth, with a correlation coefficient r value of −0.70, revealing that depth has no direct effect on the groundwater variables. The PCA results demonstrate that the GW sources of the study area are contaminated due to geological and anthropogenic processes.

Table 3 shows the Pearson correlation matrices between the groundwater variables of the study area. As an outcome, it was noticed that there were strong positive and negative correlations between the GW variables of HCO_3_^−^ and Na^+^ (r = 0.934), HCO_3_^−^ and F^−^ (r = 0.903), HCO_3_^−^ and Ca^2+^ (r = −0.607), Ca^2+^ and Na^+^ (r = −0.604), Ca^2+^ and F^−^ (r = −0.550), Mg^2+^ and SO_4_^2−^ (r = 0.407), Na^+^ and F^−^ (r = 0.815), SO_4_^2−^ and Cl^−^ (r = 0.502), and TDS and NO_3_^−^ (r = 0.523). Most of the variables support the PCA results for the GW of the study area.

### 4.6. Health Risk Assessment

The intake of fluoride-contaminated water, which has the potential to cause 80% of ailments, has a significant effect on human health. Although fluoride is recognized to be a key element in the development of human skeletal and dental enamel, elevated doses of groundwater fluoride are responsible for dental and skeletal fluorosis [58,59]. Therefore, a health risk estimation was conducted to calculate the ADD and HQ values for children and adults in the study area.

Table 4 shows the ranges of ADD_ingestion_ for children, females, and males in the Quetta Valley, which were observed to be 0.0098–0.32292, 0.007308–0.238846, and 0.00690–0.225818, with mean values of 0.093052, 0.068825, and 0.065071, respectively. The HQ_ingestion_ values ranged between 0.16467 and 5.382, 0.121795 and 3.80769, and 0.115152 and 3.763636, with average values of 1.55086, 1.147089, and 1.084521 for children, females, and males, respectively. It was noticed that children have the highest maximum and average values of ADD_ingestion_ and HQ_ingestion_ in the research area, indicating that groundwater fluoride intake poses the greatest health risk to children. The health hazards for various individuals are listed in the following order: children > females > males. Health hazards can be divided into three levels based on the HQ values: low and negligible risk (HQ < 1), medium risk (1 < HQ < 4), and high risk (HQ > 4). It has been calculated that all individuals face some level of health risk. Children, in particular, have low to high risk.

Our research suggests that groundwater monitoring and safety management are essential to meet the ever-increasing demand of population and economic activities without further deterioration of groundwater and harm to humans. Local governments should take steps to develop a regular monitoring infrastructure for the groundwater system and install safe drinking water systems.

### 4.7. Suitability Assessment of Drinking Groundwater

The water quality index (WQI) is a critical groundwater quality metric used to assess the suitability of groundwater for ingestion. The WQI is a method for calculating the impact of groundwater variables on groundwater quality, and it is calculated using the WHO’s drinking criteria. According to the WQI criteria, there are five categories: excellent (>50), good (>50), poor (>100), very poor (>200), and water unfit for drinking (>300). Table 5 depicts the WQI classification of the study region, including a list of the various types of groundwater based on the WQI references. Table 5 shows that 44% of the samples belong to the poor category, 49% are in the good category, and 8% of the samples belong to the excellent category.

## 5. Conclusions

The existence of the elevated levels of fluoride in drinking water sources may render it unsuitable for human consumption and has an adverse effect on the health of human beings. According to the study findings, 18 out of the 37 samples tested were above the WHO-recommended safe drinking limit of 1.5 mg/L. The fluoride concentrations in the tube well drinking water samples ranged between 0.19 mg/L and 6.21 mg/L, with an average value of 1.8 mg/L. The groundwater samples of the study area, 54% were of the NaHCO_3_ type, and 46% samples were of the mixed CaNaHCO_3_ type. The hydrochemistry of the groundwater is controlled by rock/water interaction. Groundwater sources of the study area were saturated with CaCO_3_ and halide minerals. The principal component analysis results reveal that the groundwater sources of the study area are contaminated due to geological and anthropogenic actions. The health risk assessment results of F^−^ show that the mean values of ADD_ingestion_ for children, females, and males in the Quetta Valley were observed to be 0.093052, 0.068825, and 0.065071, respectively. The HQ_ingestion_ mean values were 1.55086, 1.147089, and 1.084521 for children, females, and males, respectively. It was noticed that children have the highest average values of ADD_ingestion_ and HQ_ingestion_ in the research area, indicating that groundwater fluoride intake poses the greatest health risk to children. The water quality index (WQI) analysis shows that 44% of the samples in the study area belong to the poor category, 49% are in the good category, and 8% of the samples belong to the excellent category. Our research suggests that groundwater monitoring and safety management are essential to meet the ever-increasing demand of the population and economic activities without further deterioration of the groundwater and harm to humans. Local governments should take steps to build a comprehensive groundwater monitoring network and install safe drinking water wells. It is also necessary to raise public knowledge about the sustainable and safe use of groundwater resources.

## Figures and Tables

**Table 1:** Descriptive statistics of selected parameters of drinking groundwater sources.

Parameters	Minimum	Maximum	Mean	Std. Deviation	WHO Limit
pH	7.1	8.1	7.7	0.3	6.6–8.5
TDS (mg/L)	290	594	404.6	79.8	1000
EC (µs/cm)	261	705	500.3	121.6	400
Depth (feet)	70	135	96.8	15.7	-
Turbidity NTU	0.5	3.7	1.7	0.7	5
HCO_3_^−^ (mg/L)	143	532	289.5	125.6	250
Ca^2+^ (mg/L)	17	78	47.5	18.1	200
Mg^2+^ (mg/L)	15	73	30.6	13.3	150
Na^+^ (mg/L)	135	549	283.3	122.8	200
SO_4_^2−^ (mg/L)	13	90	34.9	17.5	250
NO_3_^−^ (mg/L)	0	4	1.0	0.9	10
K^+^ (mg/L)	1	4	1.6	1.0	12
Cl^−^ (mg/L)	12	89	25.6	17.3	250
Fe^2+^ (mg/L)	0.01	0.2	0.01	0.0	0.3
F^−^ (mg/L)	0.19	6.21	1.8	1.4	1.5

**Table 2:** Principal component analysis of selected parameters in the study area.

Parameters	F1	F2	F3	F4	F5
pH	0.28	**0.57**	−0.04	−0.05	−0.15
TDS	−0.24	**0.52**	**0.60**	−0.21	−0.32
EC	−0.17	**0.55**	−0.02	−0.13	0.26
Depth	0.18	−0.43	0.09	0.15	**−0.70**
Turbidity	−0.44	0.35	−0.07	**0.57**	0.00
HCO_3_^−^	**0.96**	0.08	−0.03	−0.03	0.09
Ca^2+^	**−0.68**	−0.12	0.13	0.26	0.43
Mg^2+^	0.04	−0.28	**0.54**	−0.36	0.28
Na^+^	**0.91**	0.14	−0.12	−0.11	0.12
SO_4_^2−^	0.19	−0.37	**0.81**	0.13	−0.03
NO_3_^−^	0.23	−0.44	−0.31	**0.52**	0.06
K^+^	0.29	0.43	0.11	0.33	0.16
Cl^−^	0.35	−0.11	**0.51**	0.29	0.35
Fe^2+^	−0.02	−0.38	−0.34	−0.46	0.27
F^−^	**0.92**	0.10	0.01	0.15	0.05
Total	3.75	2.00	1.84	1.34	1.20
% of Variance	24.98	13.35	12.27	8.91	8.00
Cumulative %	24.98	38.33	50.60	59.51	67.51

**Table 3:** Pearson’s correlation analysis of selected parameters.

Parameters	pH	TDS	EC	Depth	Turbidity	HCO_3_	Ca	Mg	Na	SO_4_	NO_3_	K	Cl	Fe	F
pH	1														
TDS	0.16	1													
EC	0.271	0.25	1												
Depth	−0.029	−0.065	−0.272	1											
Turbidity	−0.058	0.148	0.04	−0.187	1										
HCO_3_	0.25	−0.194	−0.099	0.048	−0.356	1									
Ca	−0.218	−0.011	0.084	−0.207	0.311	−0.607 **	1								
Mg	−0.171	0.089	−0.079	−0.076	−0.193	0.045	0.049	1							
Na	0.246	−0.235	−0.125	−0.06	−0.324	0.934 **	−0.604 **	0.05	1						
SO_4_	−0.071	0.223	−0.289	0.248	−0.197	0.13	0.056	0.407 *	0.003	1					
NO_3_	−0.058	−0.523 **	−0.092	0.182	−0.036	0.19	−0.049	−0.09	0.072	0.054	1				
K	0.245	0.09	−0.043	−0.079	0.17	0.267	0.013	−0.07	0.253	0.032	−0.125	1			
Cl	0.002	−0.024	0.114	0.043	−0.152	0.27	0.003	0.066	0.17	0.502 **	0.095	0.084	1		
Fe	−0.109	−0.308	−0.126	0.014	−0.275	−0.018	0.083	0	−0.023	−0.125	−0.047	−0.088	−0.092	1	
F	0.186	−0.168	−0.123	0.143	−0.246	0.903 **	−0.550 **	−0.035	0.815 **	0.121	0.172	0.378	0.367	−0.078	1

** Correlation is significant at the 0.01 level (2-tailed). * Correlation is significant at the 0.05 level (2-tailed).

**Table 4:** Health risk exposure assessment in the form of average daily ingestion (ADD), and hazard quotient (HQ) for children and adults consuming fluoride-contaminated groundwater of Quetta city, Pakistan (*n* = 37).

Sample ID	F (mg/L)	ADD Child	ADD Female	ADD Male	HQ Child	HQ Female	HQ Male
S1	3.62	0.188	0.139	0.132	3.14	2.32	2.19
S2	0.9	0.047	0.035	0.033	0.78	0.58	0.55
S3	1.9	0.099	0.073	0.069	1.65	1.22	1.15
S4	0.62	0.032	0.024	0.023	0.54	0.40	0.38
S5	0.72	0.037	0.028	0.026	0.62	0.46	0.44
S6	0.19	0.010	0.007	0.007	0.16	0.12	0.12
S7	4.65	0.242	0.179	0.169	4.03	2.98	2.82
S8	2.49	0.129	0.096	0.091	2.16	1.60	1.51
S9	3.65	0.190	0.140	0.133	3.16	2.34	2.21
S10	2.79	0.145	0.107	0.101	2.42	1.79	1.69
S11	1.25	0.065	0.048	0.045	1.08	0.80	0.76
S12	0.85	0.044	0.033	0.031	0.74	0.54	0.52
S13	0.67	0.035	0.026	0.024	0.58	0.43	0.41
S14	1.71	0.089	0.066	0.062	1.48	1.10	1.04
S15	0.5	0.026	0.019	0.018	0.43	0.32	0.30
S16	1.6	0.083	0.062	0.058	1.39	1.03	0.97
S17	3.92	0.204	0.151	0.143	3.40	2.51	2.38
S18	0.7	0.036	0.027	0.025	0.61	0.45	0.42
S19	1.9	0.099	0.073	0.069	1.65	1.22	1.15
S20	6.21	0.323	0.239	0.226	5.38	3.98	3.76
S21	4.51	0.235	0.173	0.164	3.91	2.89	2.73
S22	0.5	0.026	0.019	0.018	0.43	0.32	0.30
S23	1.6	0.083	0.062	0.058	1.39	1.03	0.97
S24	0.2	0.010	0.008	0.007	0.17	0.13	0.12
S25	1.4	0.073	0.054	0.051	1.21	0.90	0.85
S26	2.1	0.109	0.081	0.076	1.82	1.35	1.27
S27	1.7	0.088	0.065	0.062	1.47	1.09	1.03
S28	0.8	0.042	0.031	0.029	0.69	0.51	0.48
S29	1.7	0.088	0.065	0.062	1.47	1.09	1.03
S30	0.3	0.016	0.012	0.011	0.26	0.19	0.18
S31	1.2	0.062	0.046	0.044	1.04	0.77	0.73
S32	0.7	0.036	0.027	0.025	0.61	0.45	0.42
S33	1.56	0.081	0.060	0.057	1.35	1.00	0.95
S34	1.2	0.062	0.046	0.044	1.04	0.77	0.73
S35	1.9	0.099	0.073	0.069	1.65	1.22	1.15
S36	0.8	0.0416	0.030769	0.029091	0.69	0.51	0.48
S37	3.2	0.1664	0.123077	0.116364	2.77	2.05	1.94
Min	0.19	0.00988	0.007308	0.006909	0.16467	0.121795	0.115152
Max	6.21	0.32292	0.238846	0.225818	5.382	3.980769	3.763636
Average	1.789459	0.093052	0.068825	0.065071	1.55086	1.147089	1.084521

**Table 5:** Water quality index (WQI) classification of the study area.

WQI	Water Type	No. of Samples	% of Samples
(<50)	Excellent	3	8.10
(>50)	Good	18	48.64
(>100)	Poor	16	43.24
(>200)	Very poor	00	00
(>300)	Unsuitable for drinking	00	00

## Data Availability

Data will be provided upon request to the corresponding author.
